# Air pollution exposure during critical time periods in gestation and alterations in cord blood lymphocyte distribution: a cohort of livebirths

**DOI:** 10.1186/1476-069X-9-46

**Published:** 2010-08-02

**Authors:** Caroline EW Herr, Miroslav Dostal, Rakesh Ghosh, Paul Ashwood, Michael Lipsett, Kent E Pinkerton, Radim Sram, Irva Hertz-Picciotto

**Affiliations:** 1 Department of Public Health Sciences, University of California; Davis, USA; 2 Institute of Hygiene and Environmental Medicine, University of Giessen; Germany; 3 Bavarian Health and Food Safety Authority, Oberschleissheim; Germany; 4 Department of Genetic Ecotoxicology, Institute of Experimental Medicine, AS CR Prague, Czech Republic; 5 Department of Medical Microbiology and Immunology, University of California; Davis, CA, USA; 6 Department of Epidemiology and Biostatistics, University of California; San Francisco, CA, USA; 7 Center for Health and the Environment, University of California; Davis, CA, USA

## Abstract

**Background:**

Toxic exposures have been shown to influence maturation of the immune system during gestation. This study investigates the association between cord blood lymphocyte proportions and maternal exposure to air pollution during each gestational month.

**Methods:**

Cord blood was analyzed using a FACSort flow cytometer to determine proportions of T lymphocytes (CD3^+ ^cells and their subsets, CD4^+ ^and CD8^+^), B lymphocytes (CD19^+^) and natural killer (NK) cells. Ambient air concentrations of 12 polycyclic aromatic hydrocarbons (PAH) and particulate matter < 2.5 micrometer in diameter (PM_2.5_) were measured using fixed site monitors. Arithmetic means of these pollutants, calculated for each gestational month, were used as exposure metrics. Data on covariates were obtained from medical records and questionnaires. Multivariable linear regression models were fitted to estimate associations between monthly PAH or PM_2.5 _and cord blood lymphocytes, adjusting for year of birth and district of residence and, in further models, gestational season and number of prior live births.

**Results:**

The adjusted models show significant associations between PAHs or PM_2.5 _during early gestation and increases in CD3^+ ^and CD4^+ ^lymphocytes percentages and decreases in CD19^+ ^and NK cell percentages in cord blood. In contrast, exposures during late gestation were associated with decreases in CD3^+ ^and CD4^+ ^fractions and increases in CD19^+ ^and NK cell fractions. There was no significant association between alterations in lymphocyte distribution and air pollution exposure during the mid gestation.

**Conclusions:**

PAHs and PM_2.5 _in ambient air may influence fetal immune development via shifts in cord blood lymphocytes distributions. Associations appear to differ by exposure in early versus late gestation.

## Introduction

Ambient air pollution can affect child [1] and perinatal health [2,3]. Exposures to environmental toxicants during early life are of particular concern because developing organ systems may be especially susceptible to low-dose insults, as compared with adult life [4]. As a result, early-life exposures are more likely to produce persistent adverse outcomes [5]. However, mechanisms for such effects are less clear. It has been suggested that some aspects of air pollutant toxicity may be mediated through effects on the immune system [6].

T and B cell development start during the earlier weeks of gestation [7]. There is growing evidence that exposure to immunotoxic compounds may not only cause immuno-suppression but also result in increased expression of aberrant immune responses. A report from a workshop to identify critical windows of exposure suggests three critical phases of immune development: (1) weeks eight - 10: initiation of hematopoiesis, (2) weeks 10 - 16: hematopoietic cell migration and progenitor cell expansion and (3) weeks 16 - birth: colonization of bone marrow and thymus [8]. Toxic exposure during early gestation could result in failure of stem cell formation or abnormal formation whereas exposure in the later phases may interrupt cell migration [9] and subsequent proliferation [10]. Animals models also support the existence of differential windows of vulnerability in relation to toxicants such as lead [11].

As part of a larger investigation of the effects of air pollution on fetal and child health and development in the Czech Republic, we reported earlier that exposures to polycyclic aromatic hydrocarbons (PAH) and fine particles (< 2.5 microns, PM_2.5_) in ambient air during the 14 days prior to birth were associated with a decrease in the percentage of T lymphocytes and an increase in the percentage of B lymphocytes in cord blood [12]. We also found that greater chronic exposure to air pollution resulted in higher NK cell fractions in cord blood of newborns [13]. This report extends those findings by focusing on timing of exposures to air pollution throughout gestation and the relationship to immune markers at birth. Given that lymphocyte production, including T and B cell development, starts early in gestation [7] and that critical stages in development of the immune system may also reflect temporal variation in susceptibility to immunotoxicants, this study investigated the association between maternal exposure to air pollution during each month of gestation and cord blood lymphocyte proportions.

## Methods

The study was based in two districts of the Czech Republic, where fixed air pollution monitoring sites were established. One was in Teplice in Northern Bohemia, which is characterized by mining operations and coal-fired power plants within an area of 479 km^2^; the other was located in Prachatice in Southern Bohemia, which has light industry within an area of 1375 km^2^. The populations in Teplice and Prachatice were about 120,000 and 50,000 respectively.

### Subject enrollment and data collection

Women who delivered between May 1994 and March 1999 in the two districts were invited to participate in a pregnancy outcome study, of which about 90% (*N *= 7502) consented [14]. While in the hospital for delivery, mothers completed self-administered questionnaires regarding reproductive histories, medical conditions, medications, smoking, drinking and other lifestyle factors and occupational information. From these participants, a stratified random sample of 1,476 (about 20%) mother/infant pairs was recruited into the "Immune Biomarker Study," in which maternal and cord blood specimens were collected. Further details about recruitment and data collection have been reported previously [12].

Birth weight, gestational age, date and time of birth, length of labor, and medications administered during labor were abstracted from the medical records. Mothers with incomplete socio-demographic or labor data (*n *= 36), caesarean sections (*n *= 42) or exceptionally long labor (> 24 hours, *n *= 1) were excluded because labor has been reported to influence lymphocyte distribution [15], leaving 1,397 subjects for this study.

The study was approved by the Ethical Committee of the Regional Institute of Hygiene of Central Bohemia, Prague, and by the Human Subjects Committee of the School of Medicine at the University of California, Davis. Data and specimen collection was carried out only after obtaining written informed consent from the mothers.

### Laboratory methods: Flow cytometry

Venous cord blood sampled immediately after labor was collected into heparinized vacutainers (10 ml, Vacuette^®^, Greiner, Kremsmuenster, Austria), stored at 4°C in polystyrene boxes and transported in coolers for analysis at the Department of Genetic Ecotoxicology, Institute of Experimental Medicine, Academy of Sciences, Prague. Samples arriving at the laboratory more than 24 hours after delivery were discarded. Lymphocytes in lysed whole blood were immunophenotyped using a FACSort flow cytometer, Simulset software, and Simultest IMK lymphocyte kit of monoclonal antibodies (Becton Dickinson Immunocytometry Systems, San Jose, California, USA).

CD3^+ ^T-lymphocytes, CD3^-^CD19^+ ^B-lymphocytes, and CD3^-^CD16^+^/CD56^+ ^natural killer (NK) lymphocytes were determined. CD4^+ ^and CD8^+ ^sub-fractions of the CD3^+ ^cells were also assessed. Contamination of cord blood lymphocytes with nucleated red blood cells was resolved by a lysed whole blood method [16]. The Simulset software also provided a three-part differential of gated leukocytes based on CD45^+^CD14^+ ^staining. Corresponding percentages of identified lymphocytes (sum of T + B + NK) to the percentage of gated lymphocytes was used to control the quality of staining. The proportion of each lymphocyte subset was converted to percentages using the sum of T, B and NK lymphocytes.

### Exposure assessment

Particulate matter < 2.5 (PM_2.5_) and < 10 micrometers and PAHs were measured daily from November through March, every third day from April through June and September through October, and every sixth day in July and August. Sulfur dioxide (SO_2_), total nitrogen oxides (NO_x_), nitric oxide and nitrogen dioxide were measured daily all year round. For those days when PAHs and PM_2.5 _were not measured, they were imputed using the daily measurements of SO_2 _and NO_x_, as described previously [12].

Particle (both fine and coarse) as well as PAH concentrations were determined from samples collected by Versatile Air pollution Sampler (VAPS) [17]. The device and the measurement procedure have been described in an earlier report [12]. Twelve compounds in ambient air (phenanthrene, anthracene, fluoranthene, pyrene, benzo(a)anthracene, chrysene, benzo(b)fluoranthene, benzo(k)fluoranthene, benzo(a)pyrene (BaP), dibenzo(ah)anthracene, benzo(ghi)perylene, indeno(cd)pyrene) were measured individually and their sum was used as the total PAH levels for analysis.

In this investigation we used monthly averages of PM_2.5 _and of total PAH levels. For the exposure assessment, we assigned gestational months using the date of last menstrual period (LMP) and the date of birth. We alternated 30 and 29 day months which together with 14 days from the LMP to the estimated date of conception comprised a full term of 280 gestational days. For preterm births the exposure for the last month was averaged for the number of completed days. For example, for an infant born on eight months and ten days, the ninth month air pollution exposure was the average across the last nine days, ending the day before delivery.

### Data handling and statistical methods

All data were entered into electronic files in the Department of Genetic Ecotoxicology, Academy of Sciences, Czech Republic, Prague. One of the co-authors (MD) subsequently reviewed the data to identify and rectify errors. Outliers, implausible values and inconsistencies across variables were identified and resolved.

Gestational age in weeks was estimated by obstetricians using the LMP, number of prenatal visits, ultrasound measurements, and other information. Since gestational dating was critical to our estimates of monthly exposures, we further reviewed discrepancies between the LMP reported by the mother and that assigned based on the clinician's estimate and resolved them using birth weight, date of birth, and additional information. Prenatal care began early for most women; 93% began prenatal care and had an ultrasound examination in the first trimester of pregnancy [2].

We identified covariates of concern from the literature and developed a causal model, screening the available variables empirically, as described previously [12,13]. For instance, as reported earlier for this study population, lymphocyte distribution varied by district (higher NK cells percentages in Teplice, no notable differences in T- and B-cell percentages) and season, i.e., higher NK and lower T-cell percentages in winter and Spring in Teplice [13].

To identify potential critical time windows during gestation, we fit multivariable linear regression models to associate lymphocyte percentages with monthly average PAH and PM_2.5 _exposures, separately. We retained variables that: required adjustment to avoid confounding under the assumptions of our causal model [18], were associated with the outcome with adequate precision (p < 0.15), and resulted in changes greater than 15% in the estimated coefficient for PAHs or PM_2.5 _if removed from the model. Final models were adjusted for district and year of birth, and in further models, for season and number of prior live births. Prior pregnancy history is associated with the activity of some lymphocyte phenotypes [19] and season is strongly associated with both air pollution and lymphocyte concentrations [20]. Summer was defined as June-August; fall as September-November; winter as December-February; and spring as March-May. Because associations with fall, winter and spring were similar, we combined them into a binary variable for season of the fifth month of gestation, and assigned summer when at least 15 days of that month occurred in June-August.

We also conducted a sensitivity analysis by including average air pollution exposure in the 14 days prior to birth, in order to investigate how robust the findings were to control for short-term exposure. We categorized the short-term exposure variable to minimize potential collinearity.

Indoor use of coal and wood may markedly increase personal exposure to PAHs as can cigarette smoke [21]. Preterm or low birth weight infants were also considered to be a susceptible group with regard to immunity [22,23]. We therefore investigated possible effect modification, i.e., greater or lower associations from ambient air pollutants by exposure to smoke generated from coal or wood burning, exposure to environmental tobacco smoke, and preterm or low birth weight status.

The multivariable models were adjusted for the sampling design (stratified sampling without replacement), as described previously [12]. Results were expressed as changes in distribution of lymphocyte subtype with a monthly average increase of 100 ng/m^3 ^PAH exposure or a monthly average increase of 25 μg/m^3 ^PM_2.5_. These increments in air pollution levels were close to twice the monthly arithmetic standard deviations during the exposure period (September 1, 1993 to March 31, 1999), which were 57.3 ng/m^3 ^for PAHs and 13.5 μg/m^3 ^for PM_2.5_. This method of standardization allows comparison of the results between pollutants. The analysis was conducted in STATA statistical software, Version 10.1 (STATA, Statacorp, College Station, TX, USA).

## Results

The study sample of 1397 births was similar to the full cohort of 7502 births with respect to maternal age, maternal and paternal education, smoking and ethnicity. The randomly selected sample, however, had proportionally fewer births from the Teplice district compared to the full cohort (61% vs. 71%), more births (25%) from 1996 to 1998 (by design) and a smaller proportion of women who had three or more live births (16%) than in the full cohort (34%).

The mean proportions (SD) of the cord blood lymphocytes were 52.2 (14.2), 39.1 (11.6), 14.8 (5.7), 22 (8.3) and 25.8 (12.2) for CD3^+^, CD4^+^, CD8^+^, CD19^+ ^and NK cells respectively. The mean of the nine monthly averages for PAHs was 73.6 ng/m^3 ^and for PM_2.5_, 26.9 μg/m^3^. Similar to results reported for different time periods in these districts, mean pollutant levels were higher in Teplice (PAHs: 78.4 ng/m^3^; PM_2.5_: 31.0 μg/m^3^) than in Prachatice (PAHs: 66.4 ng/m^3^; PM_2.5_: 20.4 μg/m^3^). Because of seasonal variation in air pollution, the pollutant means were positively correlated for gestational months that were close to each other and negatively correlated if they were far apart from one another (Table 1). For instance, the mean of month one PAHs was positively correlated with the mean of month three PAHs and negatively correlated with the mean of month seven PAHs. The same was true for PM_2.5_. The correlation between the two pollutants for the same gestational month was approximately 0.8.

**Table 1:** Correlation coefficients of PAH and PM_2.5 _for the nine gestational months and 14 days prior to birth.

	PAH									PM_2.5_								
**Month**	1	2	3	4	5	6	7	8	9	1	2	3	4	5	6	7	8	9

**PAH**																		

1	1.00																	

2	0.76	1.00																

3	0.47	0.73	1.00															

4	0.07	0.44	0.73	1.00														

5	-0.29	0.06	0.47	0.75	1.00													

6	-0.53	-0.32	0.05	0.44	0.73	1.00												

7	-0.60	-0.53	-0.32	0.04	0.44	0.73	1.00											

8	-0.53	-0.56	-0.51	-0.32	0.04	0.44	0.74	1.00										

9	-0.39	-0.53	-0.56	-0.49	-0.25	0.13	0.51	0.78	1.00									

14-day	-0.33	-0.49	-0.57	-0.52	-0.33	0.02	0.40	0.67	0.88									

**PM_2.5_**																		

1	0.80	0.55	0.34	0.04	-0.25	-0.41	-0.45	-0.35	-0.23	1.00								

2	0.57	0.80	0.52	0.30	0.04	-0.27	-0.40	-0.42	-0.37	0.58	1.00							

3	0.37	0.55	0.80	0.50	0.33	0.04	-0.26	-0.35	-0.39	0.39	0.57	1.00						

4	0.11	0.36	0.55	0.80	0.51	0.30	0.02	-0.28	-0.37	0.25	0.40	0.55	1.00					

5	-0.18	0.11	0.37	0.57	0.81	0.51	0.32	0.04	-0.20	-0.01	0.25	0.39	0.56	1.00				

6	-0.35	-0.18	0.13	0.37	0.58	0.79	0.50	0.29	0.09	-0.14	0.00	0.28	0.39	0.58	1.00			

7	-0.45	-0.36	-0.18	0.12	0.39	0.57	0.81	0.51	0.36	-0.23	-0.15	-0.03	0.25	0.42	0.57	1.00		

8	-0.39	-0.40	-0.34	-0.19	0.11	0.37	0.60	0.84	0.58	-0.14	-0.21	-0.12	-0.06	0.24	0.37	0.58	1.00	

9	-0.31	-0.38	-0.39	-0.30	-0.12	0.15	0.40	0.64	0.82	-0.05	-0.16	-0.17	-0.12	0.03	0.25	0.39	0.63	1.00

14-day	-0.29	-0.39	-0.41	-0.35	-0.20	0.06	0.32	0.48	0.71	-0.18	-0.27	-0.30	-0.23	-0.15	0.08	0.26	0.37	0.72

### Regression results

#### T lymphocytes (CD3^+^, CD4^+ ^and CD8^+^)

**Figure 1:**
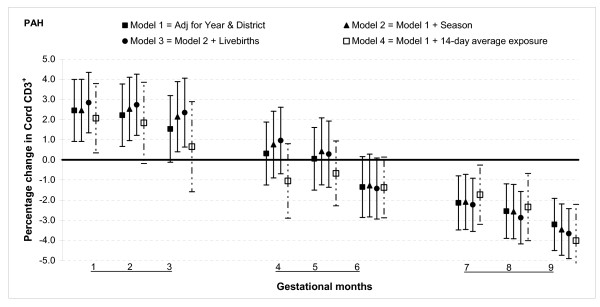
**Associations between air pollutants and CD3^+ ^lymphocyte fractions**. Percent changes in cord blood lymphocyte distributions, with 95% CI, associated with average increases of 100 ng/m^3 ^of PAH by gestational month.

**Figure 2:**
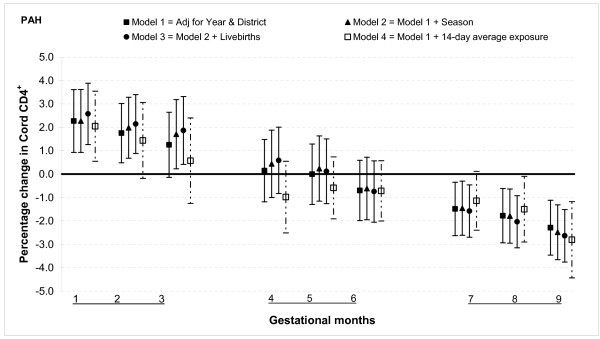
**Associations between air pollutants and CD4^+ ^lymphocyte fractions**. Percent changes in cord blood lymphocyte distributions, with 95% CI, associated with average increases of 100 ng/m^3 ^of PAH by gestational month.

**Figure 3:**
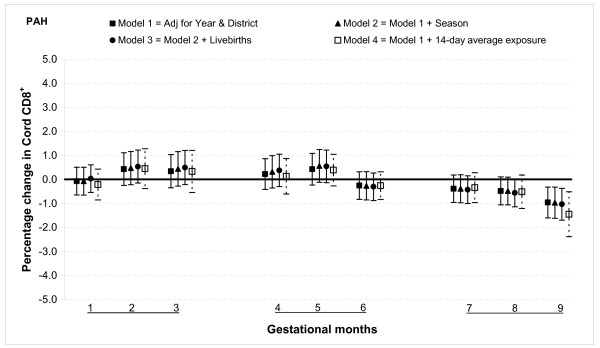
**Associations between air pollutants and CD8^+ ^lymphocyte fractions**. Percent changes in cord blood lymphocyte distributions, with 95% CI, associated with average increases of 100 ng/m^3 ^of PAH by gestational month.

The results from multivariable linear regression model adjusted for year of birth and district show that total PAH exposure during the first two months of gestation was associated with increased T lymphocyte fractions CD3^+ ^and CD4^+ ^but not with CD8^+^. For gestational month one there was a 2.5% (95% confidence interval (CI): 0.9, 4.0) increase in CD3^+ ^cells for 100 ng/m^3 ^increment in PAH (Figure 1). The magnitude was slightly smaller for CD4^+ ^cells, 2.3% (95% CI: 0.9, 3.6) for the same month (Figure 2). There was no significant association between total PAH and any T lymphocyte fractions for months three through six. However, among those with greater total PAH exposure in months seven to nine, CD3^+ ^and CD4^+ ^cell fractions were lower. For instance, a 100 ng/m^3 ^incremental increase in PAH exposure during month eight was associated with reduction of CD3^+ ^cells by 2.6% (95% CI: 1.2, 3.9) and CD4^+ ^cells by 1.8% (95% CI: 0.6, 2.9). The reduction in CD8^+ ^fractions was observed only in the ninth month: PAH exposure reduced the cell by 1.0% (95% CI: 0.3, 1.6) (Figure 3).

**Figure 4:**
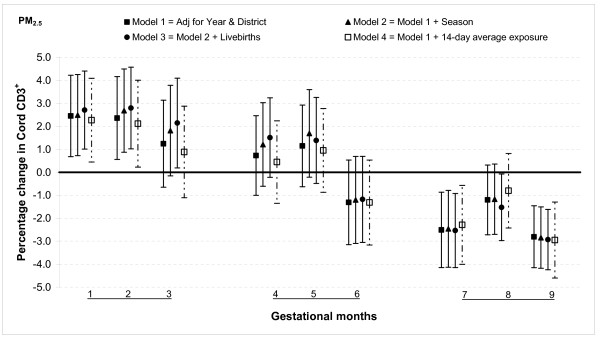
**Associations between air pollutants and CD3^+ ^lymphocyte fractions**. Percent changes in cord blood lymphocyte distributions, with 95% CI, associated with average increases of 25 μg/m^3 ^of PM_2.5 _by gestational month.

**Figure 5:**
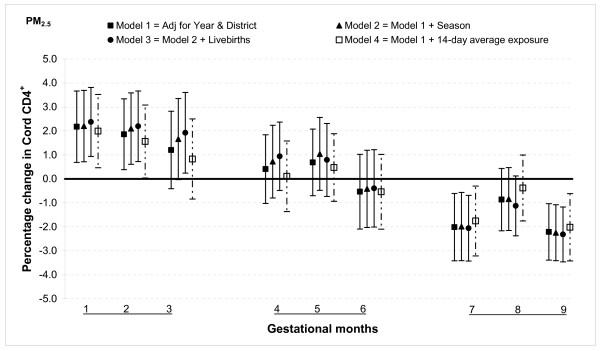
**Associations between air pollutants and CD4^+ ^lymphocyte fractions**. Percent changes in cord blood lymphocyte distributions, with 95% CI, associated with average increases of 25 μg/m^3 ^of PM_2.5 _by gestational month.

**Figure 6:**
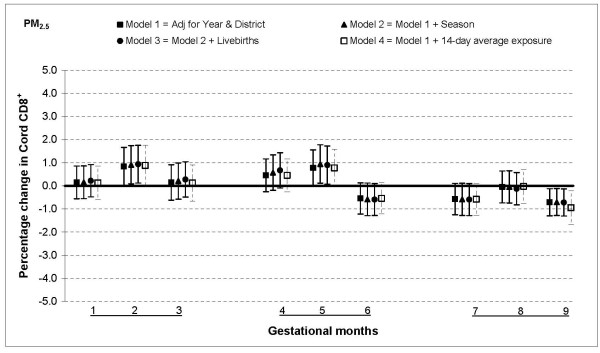
**Associations between air pollutants and CD8^+ ^lymphocyte fractions**. Percent changes in cord blood lymphocyte distributions, with 95% CI, associated with average increases of 25 μg/m^3 ^of PM_2.5 _by gestational month.

The relationship between PM_2.5 _and T lymphocytes was similar to that for PAHs: increased percentages of CD3^+ ^and CD4^+ ^cells when fine particulate exposures were higher in the first and second months of gestation, no association from months three through six and a lower percentage in months seven and nine. For instance, a 25 μg/m^3 ^increase in PM_2.5 _exposure was associated with an increase in CD3^+ ^cells by 2.5% (95% CI: 0.7, 4.2) in month one a decrease by 2.5% (95% CI: 0.9, 4.2) in month seven (Figure 4). The estimates were slightly smaller for CD4^+ ^cells, a 2.2% (95% CI: 0.7, 3.7) increase for 25 μg/m^3 ^increment in PM_2.5 _in month one and a 2.0% (95% CI: 0.6, 3.4) decrease in month seven (Figure 5). The only significant association with CD8^+ ^cells was observed in month nine when there was a 0.7% (95% CI: 0.1, 1.3) decrease for a 25 μg/m^3 ^increment in PM_2.5 _exposure (Figure 6).

#### B lymphocytes (CD19^+^)

**Figure 7:**
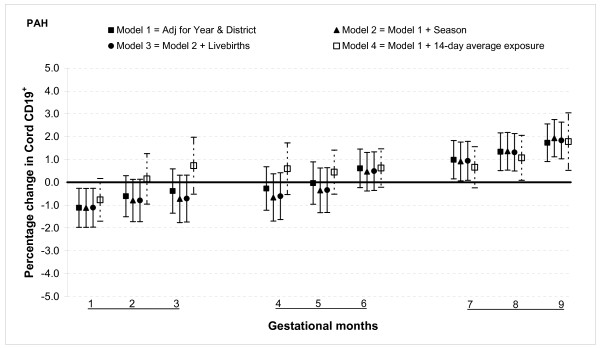
**Associations between air pollutants and CD19^+ ^lymphocyte fractions**. Percent changes in cord blood lymphocyte distributions, with 95% CI, associated with average increases of 100 ng/m^3 ^of PAH by gestational month.

The relationship between air pollution and B lymphocytes was opposite in direction to that of the T lymphocytes. The percentage of CD19^+ ^cells was lower in the first month, showed no significant association with exposures from months two through six, and increased from months seven through nine for total PAH exposure. For a 100 ng/m^3 ^increase in PAH exposure during month one was associated with a 1.1% (95% CI: 0.3, 2.0) decrease in CD19^+ ^cells (Figure 7). In the third trimester there was an increase in percentage of CD19^+ ^cells which peaked at 1.7% (95% CI: 0.9, 2.6) in month nine for a similar increase in exposure.

**Figure 8:**
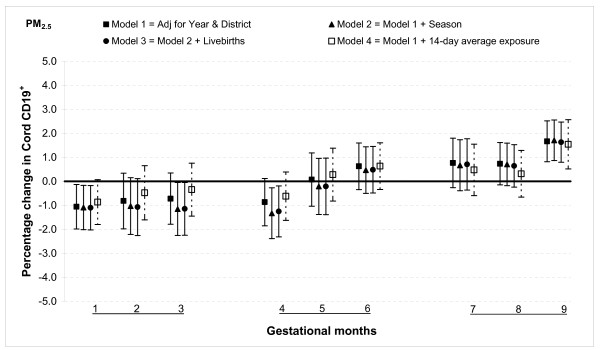
**Associations between air pollutants and CD19^+ ^lymphocyte fractions**. Percent changes in cord blood lymphocyte distributions, with 95% CI, associated with average increases of 25 μg/m^3 ^of PM_2.5 _by gestational month.

PM_2.5 _was significantly associated with this subclass of lymphocytes only in months one and nine. An increment of 25 μg/m^3 ^in PM_2.5 _was associated with a reduction in CD19^+ ^cells by 1.1% (95% CI: 0.1, 2.0) during month one, but an increase in cord CD19^+ ^cells by 1.7% (95% CI: 0.8, 2.5) in month nine (Figure 8).

#### Natural killer cells (NK cells)

**Figure 9:**
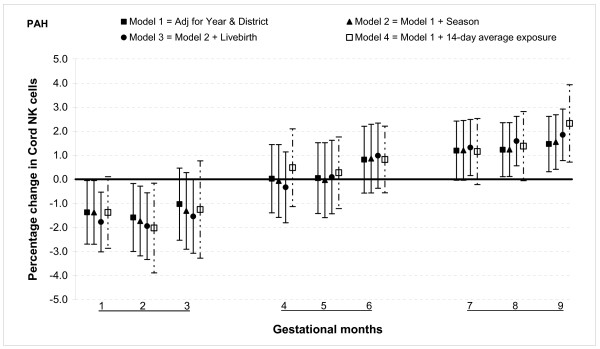
**Associations between air pollutants and Natural killer (NK) lymphocyte fractions**. Percent changes in cord blood lymphocyte distributions, with 95% CI, associated with average increases of 100 ng/m^3 ^of PAH by gestational month.

The percentage of NK cells was lower for those with higher PAH exposures in the first two months, no significant association with exposures from months three through six, and an increase in percentage relative to ambient PAHs measured during months seven through nine. For example, there was 1.4% (95% CI: 0.1, 2.7) decrease during month one and 1.2% (95% CI: 0.1, 2.4) increase during month eight for a 100 ng/m^3 ^increase in PAH exposure (Figure 9).

**Figure 10:**
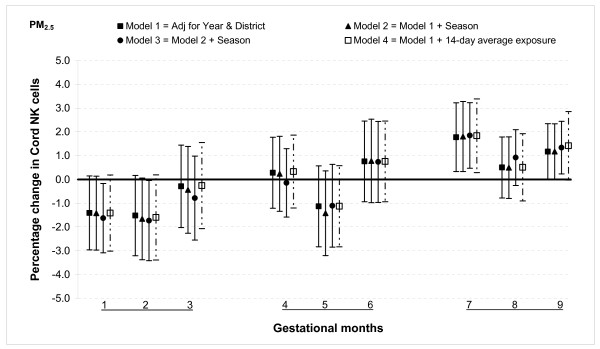
**Associations between air pollutants and Natural killer (NK) lymphocyte fractions**. Percent changes in cord blood lymphocyte distributions, with 95% CI, associated with average increases of 25 μg/m^3 ^of PM_2.5 _by gestational month.

For PM_2.5 _a significant association was observed only during month seven when 25 μg/m^3 ^increase in exposure increased the NK cells by 1.8% (95% CI: 0.3, 3.2) (Figure 10).

#### Sensitivity analysis and effect modification

In general, the associations with total PAHs were stronger than those with PM_2.5 _based on comparisons using standardized pollutant metrics (2 SDs). All of the above results were from models adjusted for district and year of birth. Further adjustment with season and number of total live births did not markedly change the associations (Figures 1, 2, 3, 4, 5, 6, 7, 8, 9 and 10). We also found no evidence of effect modification by indoor use of wood and coal, exposure to environmental tobacco smoke and preterm or low birth weight status.

## Discussion

Cord lymphocyte distributions varied with exposure to ambient pollutant levels over the nine gestational months. Overall, exposures to total PAHs or PM_2.5 _during early gestation were associated with altered distributions of lymphocyte immunophenotypes in cord blood: increases in CD3^+ ^and CD4^+ ^lymphocyte percentages and decreases in CD19^+ ^and NK cell percentages. In contrast, exposure during late gestation was associated with decreases in CD3^+ ^and CD4^+ ^fractions and increases in CD19^+ ^and NK cell fractions. There was no significant association between any of the lymphocyte distributions and either air pollutant during the middle of gestation, generally months three to six.

Studies have reported first trimester exposures to be harmful for fetal development. First trimester exposures to lead [24] or the pesticide dichlorodiphenyltrichloroethane [25] have been reported to have an adverse association with neurodevelopment and exposure to organic solvents increased the risk of congenital malformations [26]. In this study we observed that exposure during the early months of pregnancy was associated with an increase in the percentage of T cells (CD3^+ ^and CD4^+^) and a decrease in the percentage of B cells (CD19^+^), which may influence the Th1/Th2 homeostatic balance or create an environment that may lead to autoimmunity. Maintaining a Th2 skewed environment for much of the gestation is necessary for a successful pregnancy [27], but higher levels of air pollution exposure during the first trimester may alter this balance. However, this mechanism does not depend on percentages of the T helper cells alone but also on circulating cytokine levels.

The associations we observed during late gestation between lymphocyte phenotype percentages and air pollution exposure were similar to the findings we reported earlier in relation to pollutant exposures 14 days prior to birth [12] and for more chronic exposures resulting from residence in a high air pollution area [13]. Possibly the reduced T-cell percentages and increased B- and NK cell percentages are associations of more than the 14 days prior to birth, reflecting a longer period of late prenatal gestational exposure. Adjusting our models for short-term air pollution exposure (14-days prior to birth) yielded similar point estimates, but because of the correlation between the monthly and 14-day average we could not attempt for a tight control using a continuous scale. Thus, it remains uncertain whether the effects observed for the last months of gestation were confounded, with the primary impact actually arising from the last few weeks, or whether the 14-day results were confounded by associations from a somewhat longer relevant time period of late gestational exposures. In other words, the effective exposure may have been a few weeks or a few months. In either case, air pollution appears to be associated with a shift in lymphocyte production or survival based on fractions observed at birth.

Our findings indicate that timing of exposure seems to determine the direction of associations between air pollutants and lymphocyte distribution in cord blood. Alternatively, these associations might be an artifact related to seasonality in air pollution, whereby PAHs and PM_2.5 _peak in the winter and reach a nadir in the summer. The strongest negative correlations for the pollutant levels were those that were six months apart, e.g., months one vs. seven, two vs. eight,and three vs. nine. This was reflected to some extent in the magnitudes of the pollutant associations with lymphocyte proportions. For instance, PM_2.5 _and CD4^+ ^or CD3^+ ^cells showed the most extreme opposite associations in months one and seven, but months two vs. eight did not show such extremes. Had the inverse associations with lymphocytes comparing first and third trimester exposures been due to seasonality of air pollutants one might expect comparable magnitudes of associations for all these pairings.

Rather, the pattern for many associations appeared to be virtually monotonic (or close to it) over the course of gestation: PAHs and CD3^+ ^cells, PAHs and CD4^+ ^cells, PAHs and CD19^+ ^cells, PAHs and NK cells, and PM_2.5 _and CD19^+ ^cells. Thus, it is possible that the associations are truly a function of the timing of gestational exposure. This interpretation is supported by the robustness of the overall findings to adjustment for season. It also supports the notion that the previously reported finding for exposure in the 14 days prior to birth represented an association with a late gestational exposure longer than 14 days. In short, the pattern of results demonstrates that seasonality alone cannot account for the qualitatively opposite associations for first and third trimesters.

Some of the monthly air pollution averages had a moderate to high correlation. Although we fit separate models for each of the gestational months, one must be cautious not to infer that one specific month is critical while another is not. The high correlations precluded adjustment of any specific month of exposure for the surrounding months.

It has long been known that the timing of fetal exposures can affect developmental outcomes. Similar to our findings for lymphocyte distributions, a biomarker for in-utero organophosphate exposure showed the opposite associations with fetal growth when exposure occurring during four to seven weeks of gestation versus 25-28 weeks [28]. Cohen et al. reported third trimester exposure to fluoxetine (a commonly prescribed antidepressant) was associated with three-fold higher rate of newborn complications including admission to a special care nursery, compared to early exposures [29]. Research on in-utero exposure to maternal tobacco smoking has established that the strongest associations on birth weight occur with late gestational smoking, but several studies have also reported associations with early exposure [30,31]. An iron-deficient maternal environment during the first trimester, but not the second and third trimesters has been reported to be a risk factor for low birth weight [32]. To our knowledge, no previous studies have examined pollution exposures in specific time windows of gestation and immunophenotype outcomes at birth.

We examined the relative distribution of immune phenotypes with each lymphocyte subtype presented as a percentage of all cells counted and not absolute counts. If the percentage of one subfraction decreases, the percentage of at least one other will increase. Although desirable, measurement of absolute counts was not feasible due to the conditions of data collection and the need to transport samples to Prague. Nevertheless, the redistribution of lymphocyte immunophenotypes can potentially alter susceptibility to infection or to inflammatory responses. Schultz et al. reviewed life-time longitudinal patterns of lymphocytes and concluded that the developmental trajectory of absolute counts differs from that of relative distributions [33]. They further pointed out both absolute and relative sizes of lymphocyte subpopulations should be considered relevant variables for assessing maturation of the immune system.

Immunological development is intimately connected to the interactions between the organism and the environment via antigenic challenge; thus, a role for environmental pollutants in influencing such development is highly plausible. Studies have reported reduced thymus size at birth and effects on fetal immune cell counts following prenatal polychlorinated biphenyls or dioxin exposure [34,35]. In fetal mice, hypocellularity and atrophy of the thymus were associated with PAH and BaP exposure respectively [36,37], making our findings all the more relevant given that the thymus plays a critical role in differentiation, maturation and selection of T lymphocytes. Whether alterations in developmental patterns of lymphocytes have clinical implications remains to be established, but the results of our study suggest an initial link in what might be a chain of causation. Implications for childhood respiratory morbidity and atopic conditions will require further investigation.

A few data quality issues deserve consideration. Exposure data for some months, especially in the summer, had to be imputed because of the pattern of monitoring in the air-quality field measurement program, but since we focused on monthly averages, the impact of the imputations was likely to have been small. Moreover, the probability that missing data were related to the actual pollutant levels, after conditioning on daily measurements for SO_2 _and NO_x _and on the existing data for PM_10_, PM_2.5 _and PAH, is likely to be very small, supporting the assumption that these data were missing-at-random.

There was only one air monitor in operation in each district, which likely resulted in exposure misclassification. Area-wide measures of exposure to air pollution such as those obtained from fixed-site monitoring stations generally produce smaller estimates of pollutant associations compared to those determined by personal sampling [38]. Accordingly, nondifferential measurement error would tend to attenuate associations, such that the true associations may be larger than observed, but we are unable to assess the extent of this problem.

Participants of this study were randomly selected from the "pregnancy outcome study" (as described in the methods). Also, the refusal rate for this study was five percent, supporting the external validity of the results. The study sample was similar to the full cohort with respect to most of the variables; differences remaining for several variables were controlled for in the analysis through model selection and through adjustment for the sampling design by using sampling weights. Thus the results were assured generalizability to the full population. Furthermore, these results could potentially be generalized to other populations exposed to comparable levels of similar pollutants within the Czech Republic [39], Europe [40], and some cities in the USA [41].

## Conclusion

In this study, maternal exposure to ambient air pollutants during early gestation was associated with increased percentages of T lymphocytes (CD3^+ ^and CD4^+ ^cells) and reduced percentages of B lymphocytes (CD19^+^) and NK cells, whereas exposure during late gestation was associated with reduced percentages of T lymphocytes and increased percentages of B lymphocytes and NK cells. This longitudinal investigation provides evidence that exogenous factors may influence the fetal immune system in ways that depend on the timing of exposure. Early and late, rather than mid, gestation may represent critical windows of vulnerability for fetal immune development. Although the clinical consequence of such changes in lymphocyte proportions is not clear, the results suggest that the immunomodulatory actions of PAHs and PM_2.5 _may offer a plausible mechanism for some impacts of air pollutants on child and infant health.

## Abbreviations

CD: Cluster of differentiation; NK: Natural killer cells; PAH: Polycylic aromatic hydrocarbons; PM_2.5 _-Particulate matter < 2.5 microns; SO_2 _-Sulfur dioxide; NO_x _-Nitrogen oxides; VAPS: Versatile air pollution sampler; BaP: Benzopyrene; LMP: Last menstrual period; SD: standard deviations.

## Competing interests

The authors declare that they have no competing interests.

## Authors' contributions

CEWH conceptualized the analysis of specific time windows of pollution exposure in relation to lymphocyte subsets, reviewed literature during early stages of work. MD designed and oversaw the Immune Biomarker Study field work, directed the laboratory lymphocyte analyses, reviewed medical records and assembled database of lymphocyte results. RG conducted all final analyses, updated literature review, revised manuscript. PA provided immunologic expertise for interpretation of findings, reviewed final manuscript. ML provided air pollution epidemiology expertise. KP provided toxicologic expertise on PAHs and PM_2.5 _in relation to reproductive prenatal development and reviewed final manuscript. RS director of the Teplice program, obtained funding for study. IHP directed the data analysis and writing of manuscript. All authors read and approved the final manuscript.
